# Feasibility and Usability of the Job Adjustment Mobile App for Pregnant Women: Longitudinal Observational Study

**DOI:** 10.2196/48637

**Published:** 2023-11-14

**Authors:** Aya Wada, Yasuka Nakamura, Maiko Kawajiri, Yoko Takeishi, Mikako Yoshida, Toyoko Yoshizawa

**Affiliations:** 1 Physical Fitness Research Institute Meiji Yasuda Life Foundation of Health and Welfare Hachioji, Tokyo Japan; 2 Department of Women’s Health Nursing & Midwifery Tohoku University Graduate School of Medicine Sendai, Miyagi Japan; 3 Health Sciences Department of Nursing Kansai University of International Studies Miki, Hyogo Japan

**Keywords:** work, pregnancy, occupation, occupational health, mHealth, mobile application, feasibility, usability, longitudinal, observational study, pregnancy, Japan, mobile health, occupational health and safety, ergonomics

## Abstract

**Background:**

Working pregnant women often need to adjust their physically demanding jobs for a healthy pregnancy. However, uncertainty about the extent of these adjustments can hinder their effectiveness. To address this, we developed the Job Adjustment mobile app, which allows users to input job and health details to generate a variety of personalized action plans. As this is the first version of the app, assessing its feasibility and usability is crucial.

**Objective:**

This study aims to verify the feasibility and usability of the Job Adjustment mobile app.

**Methods:**

A longitudinal observational study was conducted on pregnant Japanese women who were allowed to use the app anytime from 12 to 34 weeks of gestation; they received reminder emails every 2 weeks encouraging app use. A questionnaire was administered before app use and at 20 and 32 weeks of gestation. Feasibility was evaluated across 4 domains: implementation, demand, acceptability, and adverse events. Implementation was evaluated based on 3 parameters: dropout rate, initial reminder email receipt rate, and adherence rate (measured as pregnant women who used the app at intervals of 2.5 weeks or less). Demand was measured by intervals between use and intervals between log-in, and participants answered 15 questions to assess acceptability. Adverse events were assessed by analyzing the degree of anxiety related to work. Demographic data were analyzed to determine any statistically significant differences in intervals between uses. Usability was evaluated using the System Usability Scale.

**Results:**

The analysis included 66 pregnant women, and 61% (n=40) of them were multipara. The dropout rate, adherence rate, and initial reminder email receipt rate were 18% (13/71), 44% (29/66), and 79% (52/66) respectively. The median intervals between use and intervals between log-in were 2.94 (IQR 2.00-5.13) weeks and 2.28 (IQR 1.81-4.00) weeks, respectively. Overall, 60% (35/58) to 90% (52/58) of the participants responded positively to all 15 questions assessing acceptability, and no anxiety regarding work was recorded. The mean System Usability Scale score was 66.1 points. Multipara women had significantly longer intervals between app use compared to primipara women (*P*=.01).

**Conclusions:**

The results demonstrated acceptable levels of feasibility and usability of the app. However, the low adherence rates, especially among multipara women, suggest the need for modifications to reduce the time burden of the app. Further research should explore more effective and acceptable intervals between use and timing, involving a larger sample and accounting for diverse characteristics of pregnant women.

**Trial Registration:**

UMIN Clinical Trials Registry UMIN000042943; https://tinyurl.com/ydrchfas

## Introduction

Pregnant women often need to make adjustments in their physically demanding jobs to maintain a healthy pregnancy throughout the gestation period. Pregnancy brings about physical changes in women, such as hormonal fluctuations and abdominal enlargement. Due to such physical changes, continuing to perform physically demanding jobs such as standing and lifting, as they did before pregnancy, can cause physical problems such as lower back pain and edema [1], serious pregnancy complications [2,3], and absenteeism [4]. Conversely, job adjustments during pregnancy are likely to reduce absenteeism [5]. These adjustments may involve reducing workload in the first trimester to manage hyperemesis and incorporating breaks in the third trimester while performing tasks that require standing to prevent back pain. Herein, we defined job adjustments among pregnant women, based on some previous studies [5-9], as “when a pregnant woman assesses her working conditions to determine whether her job duties affect her health in a way that constitutes a deviation from the normal course of pregnancy and then takes appropriate measured to continue working with minimal physical and mental burden.” In Japan, per the Labor Standard Law and the Equal Employment Opportunity Act, discrimination against pregnant women is legally prohibited and employers must accept work adjustments, such as light duty transfers and restrictions on overtime work, if requested by a pregnant woman. However, these adjustments are provided if the pregnant woman claims her rights from the supervisor or company. Consequently, pregnant women need support to be able to assess the need for work adjustments and to take necessary actions, such as advocating for their rights to their supervisors, when necessary.

Job adjustments during pregnancy involve 3 main components: observing work conditions and physical symptoms, assessing whether the job interferes with the normal course of pregnancy, and taking appropriate action. However, this process may not be effective if pregnant women do not have access to the necessary information and standards for making assessments. Many pregnant women continue to engage in physically demanding work [4]. A Japanese survey showed that pregnant women sought specific knowledge about types of work that need caution and the acceptable limits for standing and lifting tasks during pregnancy [10]. Therefore, in a previous study, we reviewed guidelines from across the world and clarified the job standards that can be safely adopted by pregnant women and can safely perform, with a reduced physical workload [11]. By providing these standards, we postulated that job adjustments can be promoted among pregnant women, enabling them to assess and respond to the risk level of their own jobs.

When considering action support for job adjustments, 2 crucial elements should be taken into account. First, it is important to provide multiple options for action. Several guidelines recommend that a pregnant woman’s work should not be uniformly restricted and should be adjusted according to their work and physical symptoms [1,12]. Pregnant women need not always perform their jobs within the set criteria during pregnancy but can increase or decrease their workload depending on the physical changes and workplace situation. For example, if a pregnant woman works above the standard job criteria, it is necessary to provide specific action plans and allow her the choice to stop all work activities, reduce some of the workload, or carry out her work while paying attention to any associated symptoms. The second element is an immediacy to respond to changes in physical symptoms and work. Pregnant women must choose and act immediately upon any change in symptoms or work. Assistance through a smartphone app provides this immediacy. In Japan, pregnant women can receive advice on work adjustments from their obstetrical caregivers during prenatal checkups every 2-4 weeks; however, with the use of an app, they can take action immediately without waiting for access to medical staff.

Previous studies have reported the effectiveness of various mobile health (mHealth) approaches in improving the lifestyle of pregnant women. For example, benefits such as reducing excessive gestational weight gain [13], adequate nutritional intake [14], and smoking cessation [15] were reported. However, to our knowledge, there is only 1 app that supports job adjustment for pregnant women [16]. This previous research implemented a blended care intervention, which consists of a training session provided by obstetrical caregivers and personalized advice provided via the developed app; the results revealed that more pregnant women received advice regarding work adjustments from their caregivers. Although this intervention was very valuable, it was not found to be effective in reducing workload. For pregnant women to make drastic changes in their work, they would need to involve their supervisors and the company. Herein, we have, therefore, developed a work adjustment app that provides recommendations on making work accommodations and performing self-care routines by pregnant women themselves. The app is expected to reduce absenteeism and physical and psychological symptoms during pregnancy.

However, there are several concerns with our developed app regarding feasibility and usability. Feasibility refers to whether the intervention can be provided to pregnant women according to the protocol and whether they interact and continuously participate during the intervention period [17]. This app has 2 main concerns. First, it is crucial to determine whether users will continue using the app over time. A systematic review of the feasibility of pregnant women’s apps showed that while app interventions were well received by pregnant women, some reported a high frequency of discontinued use [18]. While this issue may be of concern for apps intended to support long-term lifestyle interventions, it is not necessarily a barrier for pregnancy apps, as their use is meant for a narrow timeframe. However, there is a paucity of studies examining how intervals between uses change throughout pregnancy. It is also expected that the feasibility of the app will differ according to characteristics such as age and educational background [19]. These characteristics should be clarified for the development of more effective interventions. The second concern is specific to this app. This study is the first to report an intervention that tests the standard job criteria among working pregnant women in Japan, and it is important to examine whether these criteria may trigger work-related anxiety among them. Meanwhile, usability refers to “the extent to which a product can be used effectively, efficiently, and satisfactorily under specified conditions” [20]. There have been reports of usability issues due to inadequate information about the app [21,22]. Therefore, any newly developed mHealth app should be evaluated by the end users to clarify the degree of usability and identify usability problems [23,24]. Therefore, this study aimed to verify the feasibility and usability of the Job Adjustment mobile app.

## Methods

### Recruitment

From April 2021 to April 2022, a longitudinal observational study was conducted in Japan. The registration period was from April to September 2021. The participants were working pregnant women who satisfied the following eligibility criteria: (1) own a smartphone, (2) have no medical restrictions pertaining to work or daily life, (3) single pregnancy, (4) aged 20 years or older, and (5) being able to read and write Japanese. The exclusion criteria were women who had taken or planned to take leave before the 30th week of pregnancy. Participants were recruited by posting advertisements for recruitment and explaining the research at 7 medical facilities that handle maternity health examinations. Those who wished to participate could access the site listed in the advertisement and receive an explanation of the study from the researcher.

In terms of study size, we aimed for a minimum of 25 participants, which is considered reliable when usability is assessed by a questionnaire [25]. As this study also served as other studies to estimate the effect, a target enrollment of 75 participants was set.

### Ethical Considerations

All participants provided written informed consent. Participants who completed all surveys were given a reward worth approximately US $45, regardless of the number of times they used the app. This study was approved by the Ethics Committee of Tohoku University Graduate School of Medicine (approval 2020-1-1051).

### Intervention

#### Job Adjustment App

**Figure 1:**
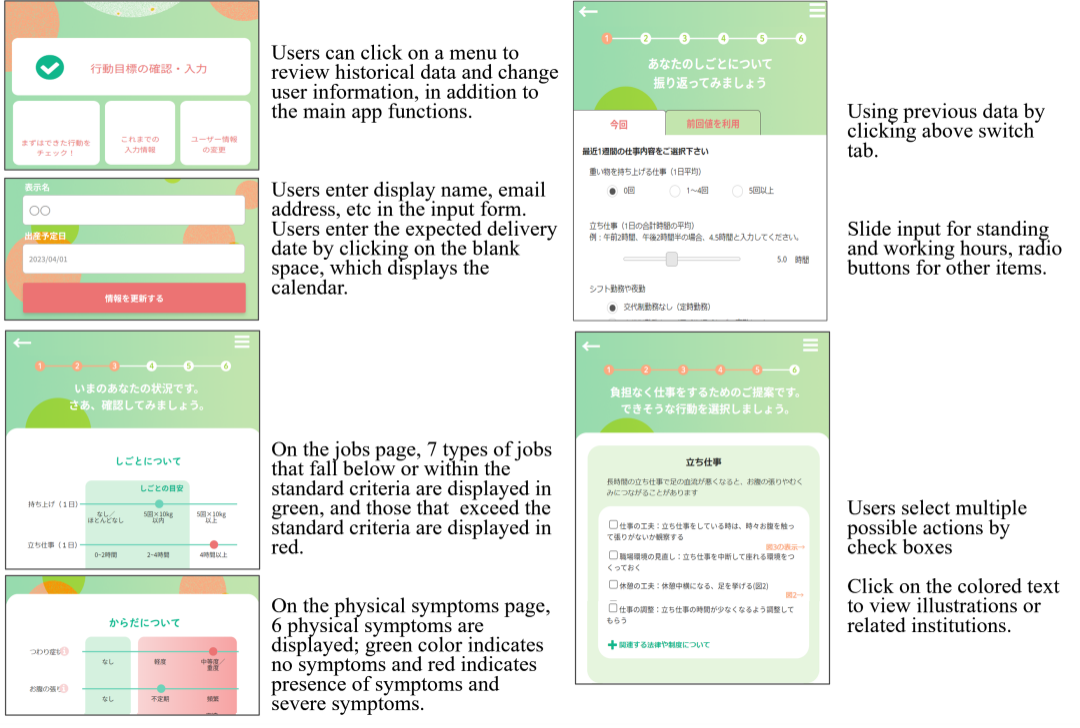
Screenshots (home setting, input stage, assessment stage and action selection stage ) of the app and user interface description.

The Job Adjustment mobile app was created as a web-based app designed for smartphones using Bubble○R , platform for the creation of digital products (Figure 1). The pregnant women who consented to research participation were registered as members of the app and could start using it after receiving a brief explanation from the researchers on how to use it.

The use of the app consisted of 3 stages: input, assessment, and action selection. At the input stage, participants were required to enter information regarding the extent or duration of their jobs from among a categorization of 7 types of jobs (lifting work, standing and sitting work, working hours, shift work, physical workload, and occupational stress) and the presence or absence and extent of 6 physical symptoms (digestive symptoms, uterine contraction, anemia symptoms, edema or constipation, low back pain, and occupational stress reaction). These items were selected based on the inclusion criteria, which were related to the job and physical symptoms and considered their potential impact on abnormal pregnancy, resignation, or the need for leave of absence; their amenability to job adjustment; and their observability by the pregnant woman herself. At the assessment stage, the app displayed the status of the input jobs and the physical condition as one of the following 3 stages: below, within, and above criteria. The job criteria were determined after reviewing the guidelines for working pregnant women worldwide [11]. This allowed the user to confirm their status at a glance and assess whether the jobs exceeded the standard criteria or if they were experiencing significant physical symptoms. At the action selection stage, the app provided multiple individual action goals for job adjustment with regard to the work and physical symptoms that exceeded the standard criteria. The action plans were developed in compliance with guidebooks and guidelines provided by public institutions or academic societies [26-29]. There are many variations of this action plan, ranging from actions that could be accomplished solely by the user to those requiring consultation with their superiors, enabling users to select the action plan that best suited their needs. The app also provided information on the laws and social systems that can support the chosen action. At the subsequent use, the users could indicate whether the selected actions had been successfully implemented and they could then input their most recent job-related and physical conditions. For example, if a pregnant woman enters that she stands for 4 hours per day at the input stage, this will be highlighted in red in the assessment stage to indicate that this is excessive. Next, in the action selection stage, 3 potential actions are proposed: “work environment: create an environment to interrupt standing and sit down,” “breaks: lie down or raise your legs/extended breaks or frequent breaks,” and “job change: make adjustments to reduce standing work.” In addition, for pregnant women who entered that they also have uterine contractions, an additional suggestion “observe symptoms: observe carefully for uterine contraction when standing” is proposed, along with a sentence emphasizing the importance of these adjustments.

Here, to streamline the process and reduce input load, the user only needed to input additional changes since the initial input. If the app use was stopped midway, the user could also start using the app from that point onward. As the first step toward usability verification, we conducted a preliminary survey of 7 working pregnant or postpartum women, identifying 33 usability issues; accordingly, we conducted this research after improving these issues. The design and interface of the app were determined in consultation with user interface and user experience experts, as well as with feedback from end users. ID-protected accounts, passwords, and all transmissions were secured by encryption.

#### Follow-Up for the Use of the Job Adjustment Mobile App

It is recommended that the Job Adjustment mobile app be used at least once every 2 weeks to prevent overlooking the changes in physical symptoms. Email reminders were delivered to users via a cloud-based email newsletter distribution system during the even-numbered weeks of pregnancy. The email content consisted of information on the general physical changes according to the number of weeks of pregnancy and the need to accordingly adjust the job duties.

We also conducted approximately 5-10-minute interviews twice while using the app. The interview aimed to confirm whether there were any troubles during use. If there was a problem, we asked about the details. Interviews were conducted approximately 4 weeks after the start of app use and at the 26th to 28th week of pregnancy. Interviews were conducted according to the participants’ preferences (either in person, via web, or by telephone). Participants for which interviews were difficult to arrange were requested to complete a web-based questionnaire.

### Data Collection Method

For the quantitative data, a URL was sent to participants’ email addresses, and the participants were requested to respond to the web-input questionnaire. The surveys were conducted 3 times: T1 at around the 12th week of pregnancy (ie, before the intervention); T2 at around the 20th week; and T3 at around the 32nd week of pregnancy. The researchers downloaded and saved the Job Adjustment mobile app use record, which was recorded on the server each time.

The qualitative data were collected by having the participants answer follow-up questions during interviews on the continued use of the Job Adjustment mobile app.

### Measurements

#### Feasibility

Feasibility was evaluated in the 4 domains of implementation, demand, acceptability, and adverse events (Multimedia Appendix 1).

Implementation was evaluated by the dropout rate, adherence rate, and initial reminder email receipt rate. The dropout rate was calculated as the percentage of registered research participants who withdrew their consent to participate. Additionally, individuals who dropped out provided their reasons for doing so on the dropout offer form. The adherence rate was determined as the percentage of those who participated until the end of the study and whose interval between app use was 2.5 weeks or less. The initial reminder email receipt rate was the percentage of the number of initial reminder emails the recipient confirmed.

The indices for demand were the interval between use and the interval between log-in of the participants, generated from the app’s log data. The intervals between use and log-in were obtained by dividing the all-use duration (weeks) by the number of times of app use and log-ins. “Use” refers to the completion of all stages offered by the app, and “log-in” indicates that the home screen has been accessed, irrespective of whether or not the features were used.

To assess acceptability, at T3, we inquired for responses to 9 items related to satisfaction, 4 items related to suitability, and 2 items related to the intention of continued use regarding the Job Adjustment mobile app, using a 4-point scale ranging from “agree” to “disagree.”

Anxiety about working was investigated as an adverse event. For this, we used an independently developed question, “How worried did you feel about your workload during pregnancy?” Responses were obtained for this question at T1, T2, and T3 on a Numerical Rating Scale ranging from 1 to 10.

#### Usability

Usability was evaluated in both quantitative and qualitative surveys (Multimedia Appendix 1). Quantitative evaluations used the System Usability Scale (SUS) score [30], obtained from surveys conducted during T2 and T3. The SUS is a widely used questionnaire for measuring the recognition of the usability of websites and smartphone apps. The survey consists of 10 questions that alternate between positive and negative questions about usability, with responses given on a 5-point scale of “strongly disagree (1)” to “strongly agree (5).” A value of 1 is subtracted from the response score for positive questions. For negative questions, the response score is subtracted from a value of 5, after which the total sum of each question score is multiplied by 2.5 to calculate the final score. The total score is 100 points, with scores <50 indicating immediate usability modification. For the qualitative evaluations, we obtained a response to the question, “What were the problems or doubts regarding the operation and input contents of the Job Adjustment Mobile App?” in interviews. This question was asked at the end of the interview, as a follow-up to the questions on the use of the app. In addition, if there were any questions about use during the initial explanation of use, these statements were also included in this analysis.

#### Participants’ Characteristics

Participants’ characteristics that were surveyed in T1 were age, birth history, educational background, job type, employment type, and smartphone use time.

#### Analysis

Descriptive statistics were used for each evaluation item for feasibility and usability. Mann-Whitney U tests and Kruskal-Wallis tests were conducted to identify differences in intervals between use in the participant characteristics. Anxiety about working was statistically tested using repeated measures ANOVA. Missing data were treated as pairwise deletion. Data analysis was conducted using SPSS (version 28.0.1; BM Software Group), with a significance level set to *P*<.05. Qualitative data about usability problems underwent participant analysis by 2 researchers and were classified into categories and subcategories. Moreover, for each subcategory, we assigned a 5-level severity based on Nielsen severity scale [31] to determine the severity of the usability problem as follows: 0=not a usability problem; 1=visual problem, fix if time permits; 2=minor usability problem, low priority to fix; 3=major usability problem, high priority to fix; and 4=critical usability problem, fixing is essential.

## Results

### Participants’ Characteristics

The participant characteristics were as follows. The average age was 33.7 (SD 3.7) years, with primipara women accounting for 39% (26/66) of all participants. Those with professional or technical occupations comprised the largest share (n=32, 48%), with full-time employees comprising 82% (n=54; Table 1). Professional and technical occupations included nurses and childcare workers, whereas sales, service, agriculture, production, and construction job types included store sales staff and factory workers.

**Table 1:** Participants’ characteristics (N=66).

Characteristics		Values
Age (years), mean (SD)		33.7 (3.7)
**Birth history, n (%)**		
	Primipara	26 (39)
	Multipara	40 (61)
**Educational background, n (%)**		
	High schools	6 (9)
	Junior college or professional school	29 (44)
	University or graduate school	31 (47)
**Job type, n (%)**		
	Professional and technical	32 (48)
	Clerical	20 (30)
	Sales, service, agriculture, production, and construction	14 (21)
**Employment status, n (%)**		
	Full-time	54 (82)
	Part-time	12 (18)
**Smartphone use time (hours per day)^a^, n (%)**		
	<2	23 (37)
	2-3	17 (27)
	>3	22 (36)

^a^With missing data (n=62).

### Feasibility

Of the 71 registered participants, 58 (82%) continued app use until T3, a total of 13 (18% [dropout rate]) dropped out by T3 due to health problems such as an imminent premature birth (n=6, 8%), 5 (7%) were untraceable, and 2 (3%) withdrew participation (one was too busy with work, and the other was unfamiliar with operating a smartphone). The 5 individuals who dropped out before T2 were excluded from the analysis, as seen in the flow chart (Multimedia Appendix 2).

In total, 66 initial reminder emails were sent, of which 5 (8%) emails resulted in an error, and the participants did not confirm 9 (14%). Consequently, the receipt rate was 79% (52/66). A different system was used in the event of an error. The emails were at times not noticed, because either the email was sent to a folder that was not in the inbox (3 emails) or the participant did not have a habit of checking her emails (6 emails). The adherence rate was 44% (29/66). The median intervals between use and intervals between log-in from the start of the app use to maternity leave were 2.94 (IQR 2.00-5.13) weeks and 2.28 (IQR 1.81-4.00) weeks, respectively. The intervals between use and intervals between log-in before and after T2 showed were 2.42 (IQR 1.67-3.67) weeks and 2.00 (IQR 1.46-3.00) weeks, respectively, from the start of use to T2; the values were 3.84 (IQR 2.28-7.00) weeks and 2.71 (IQR 2.00-7.00) weeks, respectively, from T2 to maternity leave. A comparison of intervals in terms of use by participant characteristics revealed that the app use interval was significantly longer in multipara women than that in primipara women (*P*=.01; Table 2).

**Table 2:** Differences in the interval between use by participant characteristics (N=66).

Characteristics			Participant, n (%)		Interval, median (IQR)		*P* value	
**Age group (years)**								.90^a^
	<35	37 (56)		2.88 (2.00-5.25)				
	>35	29 (44)		3.00 (2.00-5.50)				
**Birth history**								.01^a,b^
	Primipara (average age 33.5 years)	26 (39)		2.05 (1.97-4.18)				
	Multipara (average age 33.9 years)	40 (61)		4.00 (2.22-6.00)				
**Educational background**								.05^c^
	High schools	6 (9)		7.00 (4.13-8.75)				
	Junior college or professional school	29 (44)		3.00 (2.05-5.25)				
	University or graduate school	31 (47)		2.50 (2.00-4.40)				
**Job type**								.54^c^
	Professional and technical	32 (48)		2.88 (1.92-4.90)				
	Clerical	20 (30)		3.17 (2.03-5.75)				
	Sales, service, agriculture, production, and construction	14 (21)		3.44 (2.12-5.96)				
**Employment status**								.31^a^
	Full-time	54 (82)		3.25 (2.00-5.63)				
	Part-time	12 (18)		2.42 (1.85-4.21)				
**Smartphone use time (hours per day)^d^**								.74^c^
	<2	23 (37)		3.00 (2.11-5.50)				
	2-3	17 (27)		2.88 (2.00-5.40)				
	>3	22 (36)		2.80 (2.00-4.43)				

^a^Mann-Whitney U-test.

^b^*P*<.05.

^c^Kruskal-Wallis test.

^d^With missing data (n=62).

Multimedia Appendix 3 shows the results of evaluations of acceptability at T3. In response to the question, “Was this app helpful in balancing work and pregnancy?” which indicates the satisfaction evaluation, 41 (71%) out of 58 individuals gave a positive evaluation of “agree” or “slightly agree.”

The mean T1 score for anxiety about work was 5.3 (SD 2.2), the mean T2 score was 4.8 (SD 2.3), and the mean T3 score was 4.9 (SD 2.5). There was no increase in scores (*P*=.15).

### Usability

The average SUS scores were 66.08 (SD 12.34) points at T2 and 61.38 (SD 9.80) points at T3, demonstrating a decrease of 4.70 points (Multimedia Appendix 4). Usability problems were identified during the interviews (Table 3). A total of 31 usability issues were extracted and classified into 3 categories (operation, content, and display) and 7 subcategories. In addition, 3 problems were identified as level 3 problems with the highest severity: “I did not know how to input data” (including input using previous data and input of expected delivery date), “I did not know what to input as the password, display name, and action goal,” and screen display and text being cut off. Users could input data from previous entries using a button at the top that switches tabs. However, this switch tab went unnoticed by some users. The expected delivery date could be entered by clicking on the blank space in the input area, which would bring up a calendar. However, some users found this method unintuitive. Most codes were extracted during the first interview, and only the code “I am not confident that I am using the system correctly” was extracted at the second interview. Despite these issues, there were many positive comments about the home screen illustrations and the color display on the home screen illustrations and the assessment stage.

**Table 3:** Usability problems.

Severity levels^a^	Categories	Subcategories	Codes, n
3	Operation	I did not know how to input data (including input of data using previous entries and input of expected delivery date)	4
3	Content	I did not know what to input as the password, display name, and action goal	4
3	Description	Screen display and text were cut off	4
2	Content	I am unsure how to convert standing, lifting, and working hours	5
2	Content	I am unsure how to input the presence or degree of physical symptoms	3
1	Operation	I am not confident that I am using the system correctly	3
1	Operation	The slider input system is hard to use	8

^a^Higher numbers indicate greater severity [31].

## Discussion

### Principal Findings

This study attempted to verify the feasibility and usability of the job adjustment app. Our results indicate that the dropout rate was 18% (13/71), and 71% (41/58) of the pregnant women answered that “the job adjustment app helped balance work and pregnancy.” No adverse events (ie, anxiety in terms of working) were observed. The SUS score remained above 50 throughout the study. Our findings can provide valuable insights for the improvement and validation of apps designed for pregnant women, in addition to providing directions for improvement of this app.

### Feasibility

The results of the feasibility indicators of this app were similar to those of previous studies. Overdijkink et al [18] and Sakamoto et al [32] conducted a systematic review of the feasibility and effectiveness of mHealth interventions (app or SMS text message service) targeting pregnant women. Of the articles included in the review, 8 articles with intervention durations of 4 weeks or longer and 30 to 300 participants had dropout rates ranging from 6.7% to 47.6% (mean 24.7%). In the 4 articles that assessed acceptability, the percentage of pregnant women who reported that they found the intervention helpful ranged from 64.0% to 93.3% (mean 81.8%). Considering this comparison of our findings with previous research, this app is considered to have shown a certain degree of feasibility.

However, considering that the app was designed to be regularly used by pregnant women, our findings implied that the use time was not enough. In this study, only 44% (29/66) of the participants used the app once every 2 weeks, 31% (18/58) of pregnant women reported feeling burdened by the time commitment in the acceptability evaluation, and 1 pregnant woman dropped out of this research due to busy work schedule. The app required the participants to enter a total of 13 items regarding work and physical symptoms. In our preliminary study, 7 women used the app and spent an average of approximately 250 seconds at the input stage, which is longer than time spent for similar apps (175 seconds) [21]. A possible solution to this problem is to combine the app with a wearable device. In this app, extracting all the information on work and physical symptoms from only the wearable device was difficult, so we chose to have the user input everything. However, supplementing some of the input items from the wearable device may reduce the burden and encourage continued use. For instances where pregnant women’s activities were managed by a wearable wristband device, high satisfaction and wear rates have been reported [33,34]. This high acceptance rate for wearable devices in pregnant women is expected to help improve the usability of pregnancy apps in many fields.

A factor associated with feasibility, that is, “experience during childbirth,” has been demonstrated to be an important addition to factors such as educational background, age, and health literacy reported in previous studies [19]. Multipara women exhibited longer intervals between use than primipara women. It is presumed that many multipara women had high self-management abilities for adjustment of jobs due to their previous experience of balancing work and pregnancy and safely delivering a baby. As such, there is a possibility that a longer interval between app use, that is, a lower frequency of use, in this group is sufficient compared to that in primipara women. In the phase of verifying the effectiveness of this study, it is necessary to clarify the use frequencies that are suitable for both primipara and multipara women. This point is expected to be pertinent to other thematic pregnancy apps. However, to the best of our knowledge, no studies have demonstrated differences in approaches for primipara and multipara women, such as varying the intervals between use based on different birth experiences.

Despite the aforementioned improvements, we consider that this intervention was accepted by working pregnant women, indicating a need for such support. The acceptability of the app in this research exceeded 70% for almost all acceptability items. However, despite this high level of acceptability, to our knowledge, there have been only a few interventions aimed at supporting job adjustment among working pregnant women [16,35]. We must accumulate evidence in the future and develop sophisticated interventions for working pregnant women. The environment in which pregnant women find themselves plays a significant role in reinforcing the behaviors recommended by this app. To advance this field of research, we need to consider developments such as linking app data with supervisors and medical staff.

On the contrary, the low adherence rate may be owing to the recommendation to use the app once every 2 weeks being too high. Specifically, this app may be effective even in long intervals between use, and this may be especially applicable to multipara women. Several previous studies of pregnancy apps have reported no dose-response relationship between the frequency of app use or text messaging and effectiveness [36,37]. The relationship between the “interval between use” and “effectiveness” should be examined in the future.

### Usability

The job adjustment app’s usability was also verified to a certain extent. Although the SUS score in this study was slightly lower than the average score of 68 for health care apps [38], it was maintained at above 50 points. In addition, the extracted usability issues were fewer than those in previous surveys [21,39] because we had conducted a preliminary test. The iterative usability evaluation strategy appeared to be effective.

In addition, the results also provide insight into the appropriate timing for usability evaluation. Against our expectations, the usability score decreased slightly over time. We interpreted this as reflecting a natural change based on long-term use and not indicative of a usability problem. Reports of user interviews indicate that usability for any device or service reaches its highest point immediately after the start of use, then gradually declines as the freshness of the device or service wears off and slowly tends toward disposal [40]. Therefore, in our study, the usability score in the second trimester was the highest; the gradual decrease in the score did not result from a usability problem but reflected natural changes, such as the fading of freshness. This consideration is supported by the fact that most of the usability issues were identified in the initial interviews. Quantitative usability is typically evaluated across the cross-sectional study period. However, it is noteworthy that assessment performed over a long period of time after the start of use may reflect the effect of a loss of freshness to the app and may not lead to an appropriate usability evaluation.

The qualitative evaluation identified several user interface issues related to input. For example, some pregnant women had difficulty entering the expected delivery date, which was displayed in the interface as a calendar, or entering the working time by using the slider input. Therefore, in apps that require input, combining different input methods may not be recommended because it confuses the user. Nevertheless, illustrations and a screen that displayed the level of importance by color led to positive comments from pregnant women. This result is consistent with suggestions in previous studies that the use of illustrations and icons to convey information enhances usability [41].

### Limitations

This study had some limitations. First, there may have been a bias in social desirability in interviews and questionnaire responses. The study’s objectives were explained to the participants, who then participated in interviews. These participants might have, therefore, struggled to provide negative responses at this stage. Second, interviews, surveys, and rewards may have been a reminder and motivation to use the app. Because responding to all surveys was a condition for receiving rewards, some pregnant women may have used the app to complete this survey. In the future, examining the degree of demand for this app in the natural course of events will be necessary. Third, recruitment was limited; consequently, the study participants may not be representative of all working pregnant women in Japan. In Japan, of women aged 25-34 years, 26.7% work in professional occupations, and 66.7% are full-time employees [42]. In comparison, this study included a large percentage of participants who worked in professional occupations or were full-time employees, and the participants constituted a group that was in line with the need for an app that reduced their physical burdens. This may have resulted in an overestimation of values when comparing our results with those of a general population. Finally, verifying the successful implementation of job adjustments and the effectiveness of these adjustments is a future task. Despite these limitations, this study was verified longitudinally with a sample size sufficient for feasibility and usability studies [43,44].

### Conclusions

This study verified the feasibility and usability of the job adjustment app. Our findings revealed that the dropout rate, acceptability, and degree of usability were similar to those in previous studies. Our job adjustment app demonstrated a certain degree of feasibility and usability. However, multipara women had significantly longer intervals between use than primipara women. In future studies, it is necessary to verify the more acceptable intervals and time of use based on the characteristics of pregnant women.

## Appendix

Multimedia Appendix 1 Measurements and hypotheses.

Multimedia Appendix 2 Flow of study participants.

Multimedia Appendix 3 Evaluations of acceptability.

Multimedia Appendix 4 Quantitative and qualitative evaluation of usability.

## Abbreviations

mHealth: mobile health
SUS: System Usability Scale
