# Supplementary material for: Feasibility and Usability of the Job Adjustment Mobile App for Pregnant Women: Longitudinal Observational Study
Source: JMIR Form Res. 2023 Nov 14;7:e48637. doi: 10.2196/48637 (PMC10685280; doi:10.2196/48637)
Supplement: Multimedia Appendix 1 [file formative_v7i1e48637_app1.docx]

Table S1. Measurements and hypotheses.

| Domain | | | Measurement (Survey Timing) | Hypothesis |
| --- | --- | --- | --- | --- |
| **Feasibility** | | | | |
|  | **Implementation** | | | |
|  |  | Dropout rate | Percentage of registered research participants who withdrew their consent to participate | Equal to or less than that in previous studies [18,32] |
|  |  | Adherence rate | Percentage of those who participated until the end of the study and whose interval between app usage was ≤2.5 weeks (T3) | Approximately 70% |
|  |  | Initial reminder email receipt rate | Percentage of the number of initial reminder emails the recipient confirms | 100% |
|  | **Demand** | | | |
|  |  | Use interval and difference in usage intervals by attributes | “Use” indicates that all stages provided by the app have been completed. (T2, T3) | Approximately 2.5 weeks |
|  |  | Login interval | “login” indicates that the home screen has been accessed. (T2, T3) | Less than 2.5 weeks |
|  | **Acceptability** | | | |
|  |  | Percentage of positive ratings for 15 questions | 9 items related to satisfaction, 4 items related to suitability, and 2 items related to the intention of continued use (T3) | Equal to or more than in previous studies [18,32] |
|  | **Adverse events** | | | |
|  |  | Anxiety about working | The answer for independently developed question, “How worried did you feel about your workload during pregnancy?” (T1, T2, T3). Numerical Rating Scale ranging from 1 to 10 | Lower values for T2 and T3 compared to T1 |
| **Usability** | | | | |
|  | **Quantitative** | | | |
|  |  | System Usability Scale (SUS) score[ 30] | The survey consists of 10 questions that alternate between positive and negative questions about usability, with responses given on a five-point scale of “strongly disagree (1)” to “strongly agree (5).” (T2, T3) | Maintained above 50 |
|  |  | Answer for interview question | The answer for “What were the problems or doubts regarding the operation and input contents of the Job Adjustment Mobile App” (approximately 4 weeks after the start of app use and at the 26th–28th week of pregnancy) | Less than that in previous studies [21,39] |

Textbox S1.

| **Satisfaction**   1. This app was helpful in balancing work and pregnancy 2. Regular input was helpful 3. Observation items of job were helpful 4. Visualization of job was helpful 5. Standards of work duties were helpful 6. Observation items of physical symptoms was helpful 7. Visualization of physical symptoms was helpful 8. Action plans of job adjustment were helpful 9. Information on social systems was helpful   **Suitability**   1. This app covered the information on balancing work and pregnancy 2. The format of the smartphone app was easy to use 3. This app posed a time burden for me 4. This app posed a psychological burden for me   **Intention of continued use**   1. I would use this app again if I were to become pregnant again 2. I would recommend this app to working pregnant women |
| --- |
