# Supplementary figures and images for: Feasibility and Usability of the Job Adjustment Mobile App for Pregnant Women: Longitudinal Observational Study
Source: JMIR Form Res. 2023 Nov 14;7:e48637. doi: 10.2196/48637 (PMC10685280; doi:10.2196/48637)

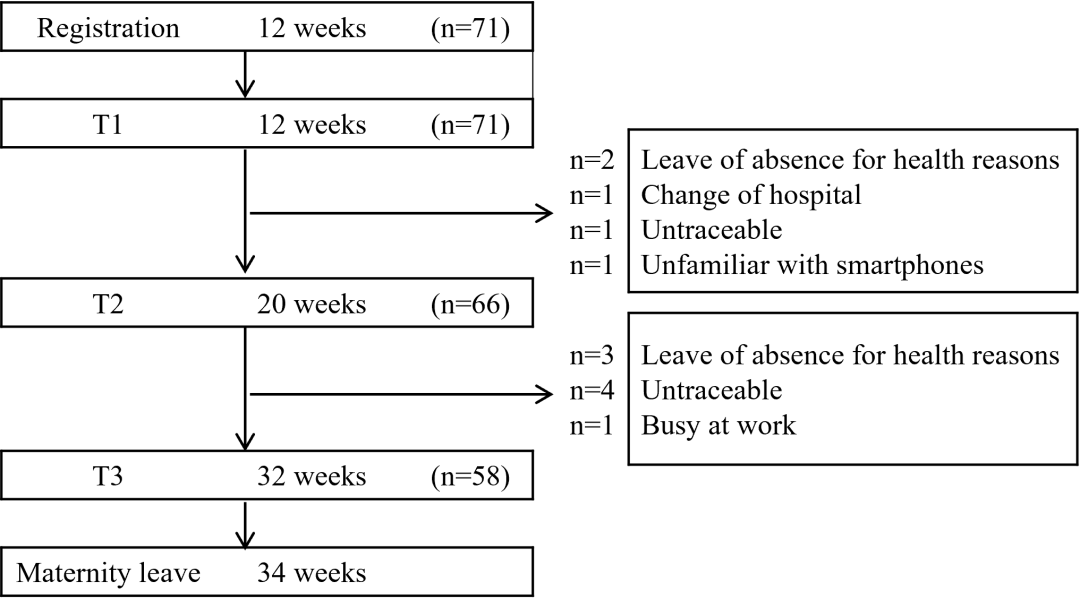

Supplement: Multimedia Appendix 2 [file formative_v7i1e48637_app2.png]

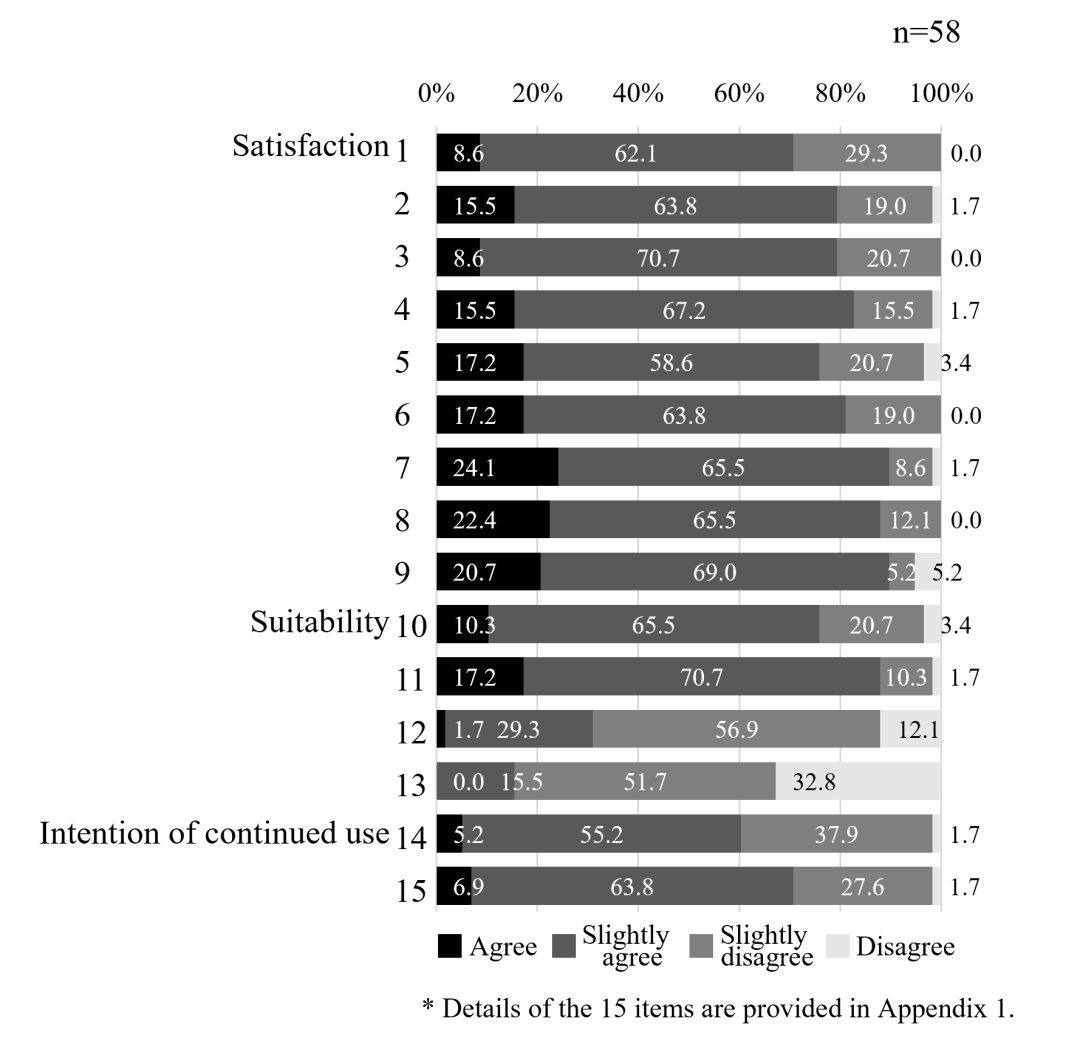

Supplement: Multimedia Appendix 3 [file formative_v7i1e48637_app3.png]

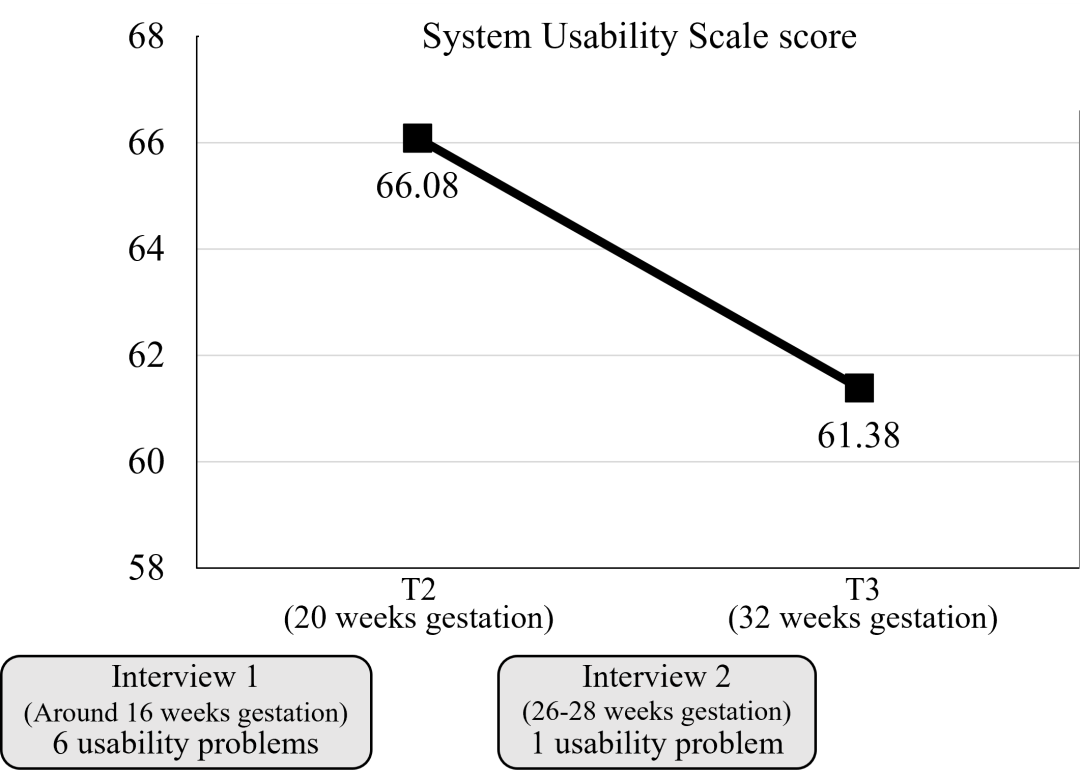

Supplement: Multimedia Appendix 4 [file formative_v7i1e48637_app4.png]
